# Phase-field modeling for pH-dependent general and pitting corrosion of iron

**DOI:** 10.1038/s41598-018-31145-7

**Published:** 2018-08-24

**Authors:** Chisa Tsuyuki, Akinori Yamanaka, Yasushi Ogimoto

**Affiliations:** 1 Department of Mechanical Systems Engineering, Graduate School of Engineering, Tokyo University of Agriculture and Technology, 2-24-16, Naka-cho, Koganei-shi, Tokyo, 184-8588 Japan; 2 Division of Advanced Mechanical Systems Engineering, Institute of Engineering, Tokyo University of Agriculture and Technology, 2-24-16, Naka-cho, Koganei-shi, Tokyo, 184-8588 Japan; 3 Advanced Technology Laboratory, Fuji Electric Co., Ltd., 1, Fuji-machi, Hino-city, Tokyo, 191-8502 Japan

## Abstract

This study proposes a new phase-field (PF) model to simulate the pH-dependent corrosion of iron. The model is formulated based on Bockris’s iron dissolution mechanism to describe the pH dependence of the corrosion rate. We also propose a simulation methodology to incorporate the thermodynamic database of the electrolyte solutions into the PF model. We show the applications of the proposed PF model for simulating two corrosion problems: general corrosion and pitting corrosion in pure iron immersed in an acid solution. The simulation results of general corrosion demonstrate that the incorporation of the anodic and cathodic current densities calculated by a Corrosion Analyzer software allows the PF model to simulate the migration of the corroded iron surface, the variation of ion concentrations in the electrolyte, and the electrostatic potential at various pH levels and temperatures. The simulation of the pitting corrosion indicates that the proposed PF model successfully captures the anisotropic propagation of a pit that is affected by the local pH of the electrolyte solution and the aggregation of Cl^−^ ions in the pit.

## Introduction

It is necessary to understand the corrosion behavior of metals to develop metals with high corrosion resistance and to maintain structural metallic components. In order to predict the corrosion behavior, various computational models were developed to simulate the morphological evolution of material surface and the diffusion–migration of ions in electrolyte solutions^1–6^. Laycock *et al*.^1^ simulated the growth of a single pit in a 304 stainless steel using the finite element (FE) method. A subsequent study revealed that the simulation results of the pitting corrosion on 304 and 316 stainless steels were in quantitative agreement with potentiodynamic experimental measurements^2^. Scheiner *et al*.^3^ developed a finite volume (FV) simulation methodology to simulate a two-dimensional evolving corrosion front in stable pitting corrosion. Onishi *et al*.^4^ developed a computational technique based on the FV method and the voxel method and analyzed time-dependent localized corrosion. Kota *et al*.^5^ proposed an FE-based simulation model to simulate the time evolutions of pit shape and the concentrations of ionic species in the electrolyte solution by solving the Nernst–Planck equation. They further applied a model to simulate stable pit growth in a polycrystalline microstructure in a SUS316 stainless steel. Brewick *et al*.^6^ also developed a FE-based simulation model for simulating the pit growth behavior that depends on crystallographic orientation of the polycrystalline microstructure in 316 L SS austenitic stainless steel. Yin *et al*.^7^ reported FE-based modeling to simulate the micro-galvanic corrosion of Al alloys where the Al dissolution rate depends on pH of the electrolyte solution. In order to consider the pH-dependent corrosion kinetics, the model incorporates the chemical-dependent current densities obtained by experiments as input data. Although the previous FE and FV-based models successfully simulated the corrosion processes, the models require explicit tracking of a moving interface between metals and electrolyte solutions to apply boundary conditions to the interface. Thus, the numerical simulations using the aforementioned models allow us to perform computationally expensive re-meshing and mesh refinement.

Conversely, the phase-field (PF) method recently attracted attention as a powerful computational method that simulates a spatiotemporal evolution of material surface driven by electrochemical reactions. The PF method was originally developed as a computational methodology to simulate microstructure evolutions in solidification^8,9^, grain growth^10,11^, and solid phase transformations^12–16^ in materials without the explicit tracking of moving grain boundaries and solid/liquid interfaces. As a pioneering study on the application of the PF model to simulate electrochemical reactions, Guyer *et al*.^17^ formulated the PF model that captures the formation of interfacial double layer in an electrochemical system. They also applied a PF model for electrodeposition and electrodissolution processes^18^. Subsequently, a few PF models that describe the stress evolution, the ionic diffusion and the interfacial migration in corroding materials were reported^19–22^. Stahle *et al*.^19^ constructed a PF model to simulate both pit growth and crack growth driven by stress corrosion cracking. Mai *et al*.^20^ proposed a PF model to simulate pitting corrosion in metallic materials and suggested that their model could simulate the evolutions of pits in the polycrystalline stainless steel and the ionic concentration in the solution. In a subsequent study, Mai *et al*.^21^ extended the PF model to simulate the stress corrosion cracking behavior in a polycrystalline microstructure and the galvanic corrosion processes^22^.

The above-mentioned PF models were developed to simulate various corrosion processes. However, to the best of the author’s knowledge, a PF model to simulate the corrosion behavior of iron and steels that depends on the pH of electrolyte solutions was not proposed in extant studies. Specifically, the experimental measurements by Bockris *et al*.^23^, Kelly *et al*.^24^, and Keddam *et al*.^25,26^ already reveal that the dissolution rate of iron is strongly affected by the pH of electrolyte solutions. Additionally, the rate of the pitting corrosion significantly depends on the pH value of the electrolyte solution in the growing pit. Therefore, the development of a PF model that enables the simulation of the pH-dependent corrosion process is essential to predict the practical corrosion behaviors in a more quantitative manner.

Furthermore, with respect to various input parameters and data required to numerically simulate the corrosion, the thermodynamic data of electrolyte solutions constitutes extremely essential data to simulate the aqueous corrosion behavior quantitatively. For example, Corrosion Analyzer^27^ is a commercial software developed by OLI Systems Inc. and provides thermodynamic data of electrolyte solutions, such as corrosion potential, anodic and cathodic current densities, with respect to various temperatures, electrostatic potentials, chemical compositions of alloys, and pH values of electrolyte solutions. Therefore, it is necessary to develop a simulation methodology to incorporate this type of thermodynamic data of electrolyte solutions into the PF model.

The present study proposes a new PF model to simulate the pH-dependent corrosion processes in ferrous metals. The PF model is formulated based on the Bockris mechanism^23^ that is among the most widely accepted iron dissolution mechanisms that consider the pH-dependence of the iron dissolution. We also propose a methodology to incorporate the thermodynamic data of electrolyte solutions obtained by Corrosion Analyzer software into the PF model. In this study, we perform the numerical simulations of two typical corrosion problems by using the proposed PF model, namely general corrosion and pitting corrosion in a pure iron immersed in an acid solution. We examine these applications of the proposed PF model and demonstrate that the PF model captures the key features of the corrosion processes including the spatiotemporal migration of the corroded iron surface, evolutions of ionic concentrations, and local pH in the electrolyte solution, and the electrostatic potential during general and pitting corrosions.

## Electrochemical reactions in corrosion of iron

In the corrosion of an iron in an electrolyte solution, the anodic and cathodic reactions denote the dissolution of iron electrode and the reduction of H^+^ irons, respectively. These reactions are expressed as follows:

1 $${\rm{Fe}}\to {{\rm{Fe}}}^{2+}+2{{\rm{e}}}^{-}$$

2 $$2{{\rm{H}}}^{+}+2{{\rm{e}}}^{-}\to {{\rm{H}}}_{2}$$

In contrast, as Kelly *et al*.^24^ reported, the iron dissolution in an acid solution depends on the pH of the electrolyte solution. One of the most well-known mechanisms of the pH-dependent iron dissolution is Bockris’s mechanism^23^. The electrochemical reactions proposed by Bockris *et al*. are expressed as follows:

3 $${\rm{Fe}}+{{\rm{OH}}}^{-}\to {({\rm{FeOH}})}_{{\rm{ads}}}+{{\rm{e}}}^{-}$$

4 $${({\rm{FeOH}})}_{{\rm{ads}}}\to {{\rm{FeOH}}}^{+}+{{\rm{e}}}^{-}$$

5 $${{\rm{FeOH}}}^{+}+{{\rm{H}}}^{+}\leftrightarrows {{\rm{Fe}}}_{{\rm{aq}}}^{2+}+{{\rm{H}}}_{2}{\rm{O}}$$

Equation (3) denotes that an iron atom combines with a OH^−^ ion in the solution to form the adsorbed intermediate Fe(OH)_ads_. Subsequently, as expressed in Eq. (4), Fe(OH)_ads_ decomposes into a FeOH^+^ ion and an electron, e^−^. The FeOH^+^ ion is oxidized by a H^+^ ion, and a Fe^2+^ ion is generated. It should be noted that the PF model proposed in this study is formulated on the basis of the electrochemical reaction given by Eq. (4) is the rate determining process of iron dissolution.

In the pitting corrosion, the iron dissolution is localized at the iron electrode surface where the protective passive film is destroyed^28^. Due to the localized dissolution of iron, a pit initiates and propagates into the iron electrode. In the propagating pit, a Fe^2+^ ion reacts with H_2_O as follows:

6 $${{\rm{Fe}}}^{2+}{+2{\rm{H}}}_{2}{\rm{O}}\to {\rm{Fe}}{({\rm{OH}})}_{2}+2{{\rm{H}}}^{+}$$

Equation (6) indicates that the pH of the solution inside the pit decreases due to the production of H^+^ ion when the pit grows. Conversely, Cl^−^ ions migrate from the bulk electrolyte solution into the pit to maintain the charge neutrality, and thus the concentration of Cl^−^ ion in the pit increases with the propagation of the pit. Furthermore, the formation and the hydrolysis of iron chloride (FeCl_2_) occurs in the pit, and this causes the formation of H^+^ ion and the reduction of pH in the pit. The reduction of pH in the pit leads to the continuous growth of the pit. Therefore, the key features of the pitting corrosion that are needed to be simulated by the PF model denote the pH-dependent iron dissolution, the reduction of the pH, and the increase in Cl^−^ ion concentration in the pit.

## Phase-field model

This section outlines the formulation of the PF model proposed in this study. (See the Supplemental file for the details of the formulation.) Although the PF model in conjunction with the Corrosion Analyzer software is applied to simulate general corrosion and pitting corrosion on a pure iron immersed in various aqueous solutions, the PF model is presented to simulate the corrosion processes in a pure iron in an acidic solution that contains Fe^2+^, H^+^, OH^−^, and Cl^−^ ions.

In order to describe the migration of the corroding iron surface, the phase-field variable, *ξ* (***r***, *t*), is defined as the local existence probability of the iron electrode, where ***r*** and *t* denote the coordinate and the time, respectively, *ξ* (***r***, *t*) corresponds to 0 in the electrolyte solution and 1 in the iron electrode, and *ξ* (***r***, *t*) changes continuously from 0 to 1 across a diffuse interface between the electrolyte solution and the electrode. The spatiotemporal evolution of the phase-field variable implicitly describes the migration of interface during the corrosion process. Additionally, *c*_i_ (***r***, *t*) (i = Fe^2+^, H^+^, OH^−^ and Cl^−^) is defined as the concentration of ionic species i in the electrolyte solution. Hereafter, (***r***, *t*) is not expressed for a simple description.

The total free energy of the system is defined by the Ginzburg–Landau free energy functional as follows:

7 $$G={\int }_{V}[{g}_{{\rm{dble}}}(\xi )+{g}_{{\rm{act}}}(\eta ,\xi )+{g}_{{\rm{grad}}}(\nabla \xi )+{g}_{{\rm{elec}}}\,(\overrightarrow{c},\,\varphi )],$$

where $\overrightarrow{c}$ = {*c*_Fe2+_, *c*_H+_, *c*_OH−_, *c*_Cl−_} denotes the concentrations of Fe^2+^, H^+^, OH^−^ and Cl^−^ ions. Additionally, *g*_dble_(*ξ*) denotes the double-well potential energy density describing an energy barrier between the following two states: the electrolyte solution (*ξ* = 0) and the iron electrode (*ξ* = 1), and *g*_dble_(*ξ*) is given as follows:

8 $${g}_{{\rm{dble}}}(\xi )=W{\xi }^{2}{(1-\xi )}^{2},$$

where *W* denotes the barrier height of the double-well potential energy, and it is expressed as follows:

9 $$W=\frac{6\gamma b}{\delta },$$

where *b* is a constant given by *b* = 2tanh^−1^(1 - 2*λ*), *λ* is a constant that defines the interfacial region as *λ* ≤ *ξ* ≤ 1 −*λ*, and *λ* = 0.1 is often employed. Furthermore, *g*_act_(*η*, *ξ*) denotes the density of activation energy density and is expressed as follows:

10 $${g}_{{\rm{act}}}(\eta ,\,\xi )=h(\xi )zF\eta ,$$

where *η* is the overpotential of the electrochemical reaction given by Eq. (4). Although the overpotential consists of the activation, *η*_a_, and concentration overpotentials, *η*_c_, we approximate *η* ≈ *η*_a_ (see Supplemental information for detailed derivation of the overpotentials). *z* denotes the number of electrons involved in the electrochemical reactions on the iron electrode surface, and *F* denotes Faraday’s constant. Additionally, *h*(*ξ*) is an interpolating function that is expressed as follows:

11 $$h(\xi )={\xi }^{2}(3-2\xi ).$$

The sum of *g*_dble_(*ξ*) and *g*_act_(*η*, *ξ*) represents the local free energy density of the iron electrode and the electrolyte two-phase mixture, *g*_grad_(∇*ξ*) denotes the gradient energy density that corresponds to the excess free energy due to the existence of interface between the iron electrode and the electrolyte solution, and *g*_elec_($\overrightarrow{c}$, *ϕ*) denotes the electrostatic energy density. The energy densities are given as follows:

12 $${g}_{{\rm{grad}}}(\nabla \xi )=\frac{1}{2}\kappa {\nabla }^{2}\xi $$

13 $${g}_{{\rm{elec}}}(\overrightarrow{c},\,\varphi )=F{\sum }_{i}{z}_{i}{c}_{i}\varphi \,({\rm{i}}={{\rm{Fe}}}^{2+},\,{{\rm{H}}}^{+},\,{{\rm{OH}}}^{-},\,{\rm{Cl}}),$$

where *z*_i_ denotes the valance of ionic species i (i = Fe^2+^, H^+^, OH^−^, and Cl^−^), and *ϕ* denotes the electrostatic potential. Furthermore, *κ* denotes the gradient energy coefficient and is given as follows:

14 $$\kappa =\sqrt{\frac{3\delta \gamma }{b}},$$

where *γ* denotes the interfacial energy and *δ* denotes the thickness of diffuse interface.

Based on the nonlinear PF model for the electrochemical systems proposed by Liang *et al*.^29,30^ and Chen *et al*.^31^, we obtain the following time evolution equation of the phase field variable:

15 $$\frac{\partial \xi }{\partial t}=-\,{L}_{\sigma }(\frac{\partial {g}_{{\rm{dble}}}(\xi )}{\partial \xi }-\kappa {\nabla }^{2}\xi )-{L}_{\eta }L(\xi )\{{K}_{1}{a}_{{{\rm{OH}}}^{-}}\exp [(1-\alpha )(\frac{zF{\eta }_{a}}{RT})]-\frac{{a}_{{{\rm{Fe}}}^{2+}}{a}_{{H}_{2}O}}{{K}_{3}{a}_{{H}^{+}}}\exp [-\alpha (\frac{zF{\eta }_{a}}{RT})]\},$$

and

16 $$L(\xi )=6\xi (1-\xi ).$$

where *L*_*σ*_ denotes the mobility of the interface between the iron electrode and the electrolyte that is moved by the gradient and the double-well potential energies. *L*_*η*_ denotes the mobility of the moving interface driven by the electrochemical reaction, and *α* denotes the transfer coefficient. Τhe activation overpotential, *η*_*a*_, is given by *η*_a_ = *E* – *E*_eq_ where *E* denotes the applied potential of the electrode. Additionally, *E*_eq_ represents the equilibrium potential of the iron electrode in the electrochemical reactions given by Eqs (3–5) (see Eq. (S5) in Supplemental information for the detail derivation). In this study, *E*_eq_ is evaluated as a function of the pH, ion concentration in the electrolyte, and temperature by using the Corrosion Analyzer software. Furthermore, *K*_1_ and *K*_3_ denote the equilibrium constants of the chemical reactions as expressed by Eqs (3) and (5), respectively (see Eqs (S11) and (S12) for the detail formulation). *a*_i_ represents the activity of ionic species i (i = Fe^2+^, OH^−^, H_2_O and H^+^). *L*(*ξ*) is used given that the iron dissolution occurs only in the interfacial region. Equation (15) describes the migration of the corroding iron surface that depends on the H^+^ ion concentration and the pH in the electrolyte solution which is calculated by pH = - log*c*_H+_.

The time evolution of the ion concentration in the electrolyte is expressed by the Nernst–Planck equation that comprises of the diffusion of ions by the concentration gradient and the migration of charged ions by the electrostatic-field as follows:

17 $$\frac{\partial {c}_{{\rm{i}}}}{\partial t}=\nabla \cdot ({D}_{{\rm{i}}}^{{\rm{eff}}}\nabla {c}_{{\rm{i}}})+\nabla \cdot (\frac{{D}_{{\rm{i}}}^{{\rm{eff}}}{c}_{{\rm{i}}}}{RT}{z}_{{\rm{i}}}F\nabla \varphi )+{I}_{{\rm{i}}}\,({\rm{i}}={{\rm{Fe}}}^{2+},\,{{\rm{H}}}^{+},\,{{\rm{OH}}}^{-},\,{\rm{Cl}}),$$

where *D*_i_^eff^ denotes the effective diffusion coefficient of the ionic species i that is given as follows:

18 $${D}_{{\rm{i}}}^{{\rm{eff}}}=\xi {D}_{{\rm{i}}}^{e}+(1-\xi ){D}_{{\rm{i}}}^{{\rm{s}}},$$

where *D*_i_^e^ and *D*_i_^s^ denote the diffusion coefficients in the iron electrode and the aqueous solution, respectively. Specifically, *I*_i_ in Eq. (17) describes the rate of production or consumption of the ionic species i (i = Fe^2+^, H^+^, OH^−^ and Cl^−^) by the electrochemical reactions on the iron surface. We assume that the variations of OH^−^ and Cl^−^ ion concentrations do not affect the corrosion rate compared to the pH of the electrolyte solution, and thus only the production of Fe^2+^ ion and the consumption of H^+^ ion are calculated.

We assume that the charge neutrality in the electrolyte solution, the distribution of the electrostatic potential in the electrolyte is given by solving the following Poisson-type equation:

19 $$\sigma {\nabla }^{2}\varphi =-\,{z}_{{{\rm{Fe}}}^{2+}}F{\rho }_{{\rm{Fe}}}{\rm{\Delta }}\xi ,$$

where *σ* denotes the electric conductivity of the electrolyte and *ρ*_Fe_ denotes the molar density of iron. Additionally, *Δξ* denotes the variation of the phase field variable representing the amount of the dissolved iron electrode. As assumed by Mai *et al*.^21^, the interfacial double layer is not considered in the study because the thickness of the interfacial double layer is in the order of nanometers. If we consider the double layer at the diffuse interface used in the PF model, it is necessary to use a very fine mesh with dimensions lower than a nanometer and a small time increment in the numerical simulation. Thus, it is impossible to perform the PF simulations of the practical corrosion processes in realistic time and length scales.

## Results and Discussion

In this section, we present the applications of the proposed PF model to the simulations of the general corrosion and the pitting corrosion on a pure iron immersed in an acid solution. Initially, we show the results of one-dimensional (1D) simulation of the general corrosion. The 1D simulation of the general corrosion is used to examine whether the PF model can simulate the corrosion process that depends on the pH of the electrolyte solution and temperature. Subsequently, the three-dimensional (3D) simulation of the pitting corrosion is presented to demonstrate the applicability of the proposed PF model to a more complex corrosion process. In the simulations, we assume that the bulk pH of the electrolyte solution and the Cl^−^ concentration are controlled by adding HCl and NaCl, respectively. The details of the conditions used in the simulations are described in Methods.

### General corrosion

**Figure 1:**
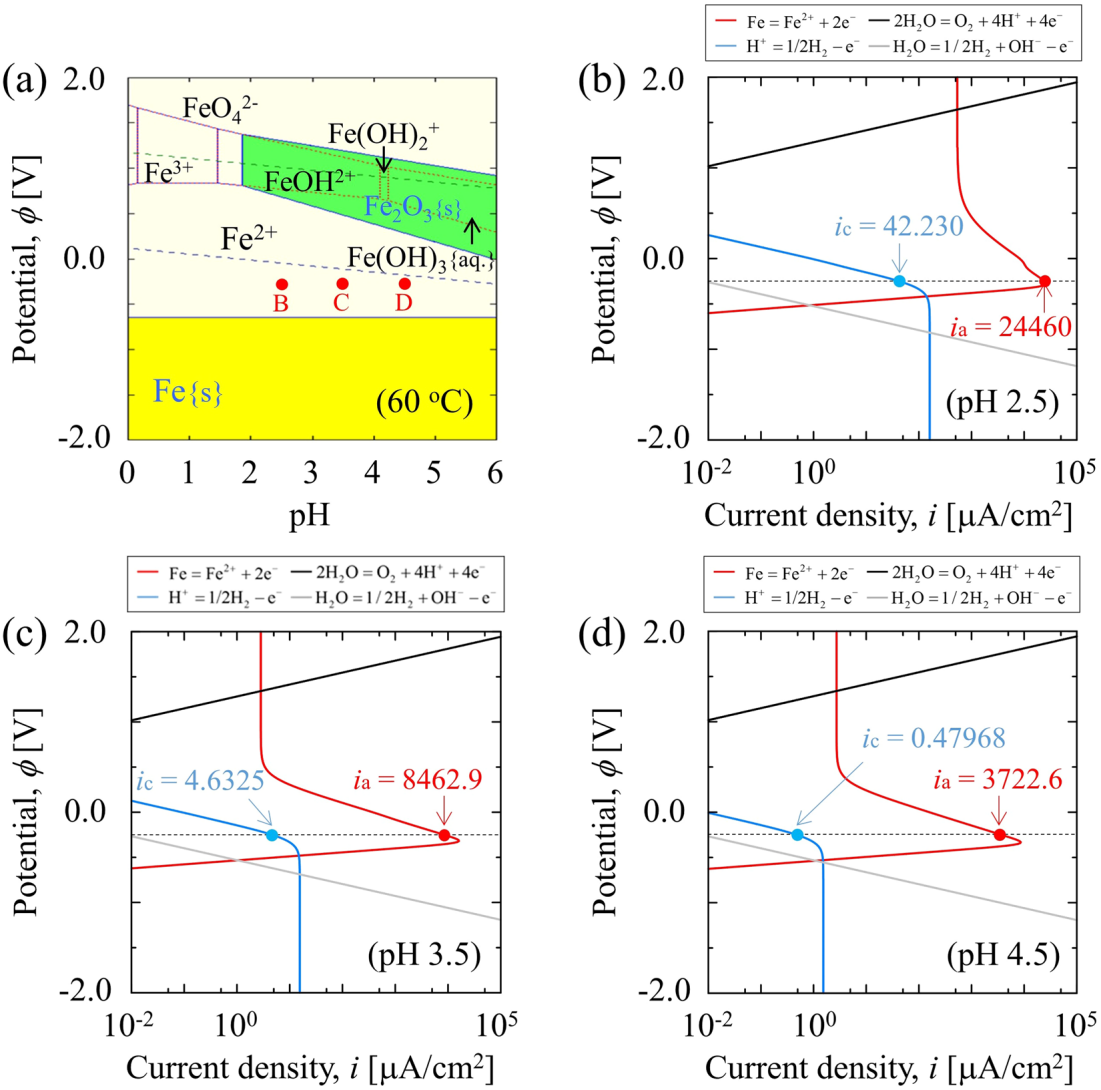
(**a**) Pourbaix diagram at 60 °C and polarization curves at the pH of (**b**) 2.5, (**c**) 3.5, and (**d**) 4.5 as calculated by Corrosion Analyzer software. (**b**,**c**), and (**d**) show the polarization curves at each pH indicated by B, C, and D in (**a**), respectively. The anodic and cathodic current densities, i.e., *i*_a_ and *i*_c_, are inserted on the polarization curves.

In the simulations of the general corrosion, five different cases are investigated. (See Table 1 in Methods.) In all cases, the applied potential of the iron electrode is assumed as constant at *E* = −250 mV vs. SHE. As an example, Fig. 1(a) shows the Pourbaix diagram for a pure iron at 60 °C as calculated by Corrosion Analyzer software. Based on the Pourbaix diagram, the general corrosion of pure iron is expected to occur in the electrolyte solution with pH corresponding to 2.5, 3.5, and 4.5. As shown in Fig. 1(b–d), the Corrosion Analyzer software also provides the anodic and cathodic polarization curves at the aforementioned pH levels. By using these current densities, *I*_i_, in Eq. (17) is calculated. (See Methods for the details of the current densities used in this study.)

**Table 1:** Physical values and parameters used in the study.

Symbol	Value
Interfacial energy, *γ* [J/m^2^]	0.5
Transfer coefficient of the Butler–Volmer kinetics, *α*	0.5
Diffusion coefficient of Fe^2+^ ion in the electrolyte^27^, ${D}_{{{\rm{Fe}}}^{2+}}^{{\rm{s}}}$ [m^2^/s]	1.52 × 10^−9^
Diffusion coefficient of H^+^ ion the electrolyte^27^, ${D}_{{{\rm{H}}}^{+}}^{{\rm{s}}}$ [m^2^/s]	15.0 × 10^−9^
Diffusion coefficient of OH^−^ ion the electrolyte^27^, ${D}_{{{\rm{OH}}}^{-}}^{{\rm{s}}}$ [m^2^/s]	9.0 × 10^−9^
Diffusion coefficient of Cl^−^ ion the electrolyte^27^, ${D}_{{{\rm{Cl}}}^{-}}^{{\rm{s}}}$ [m^2^/s]	4.0 × 10^−9^
Diffusion coefficient of all ion in iron electrode, *D*^e^ [m^2^/s]	0
Equilibrium constant for Bockris kinetics (Eq. (3)), *K*_1_	1.0
Equilibrium constant for Bockris kinetics (Eq. (5)), *K*_3_	1.0
Conductivity of the electrolyte^20^, *σ*^s^ [S/m]	1.0
Equilibrium potential^27^, *E*_eq_ [mV]	− 540

As shown in Fig. 1(b–d), the current densities strongly depend on the pH of the electrolyte solution while the pH-dependence of other parameters, material constants, and the equilibrium potentials is considered as negligible. We use the parameters and the material constants listed in Table 1, and they are assumed as independent of the pH of the solution. The diffusion coefficients of ions in the electrolyte solution and the equilibrium potential are calculated by Corrosion Analyzer software. The equilibrium constants for Bockris’s mechanism, i.e., *K*_1_ and *K*_3_, were not obtained, and thus we use these constants as fitting parameters. Unless stated otherwise, the parameters and constants shown in Table 1 are used for simulations of both general corrosion and pitting corrosion.

**Figure 2:**
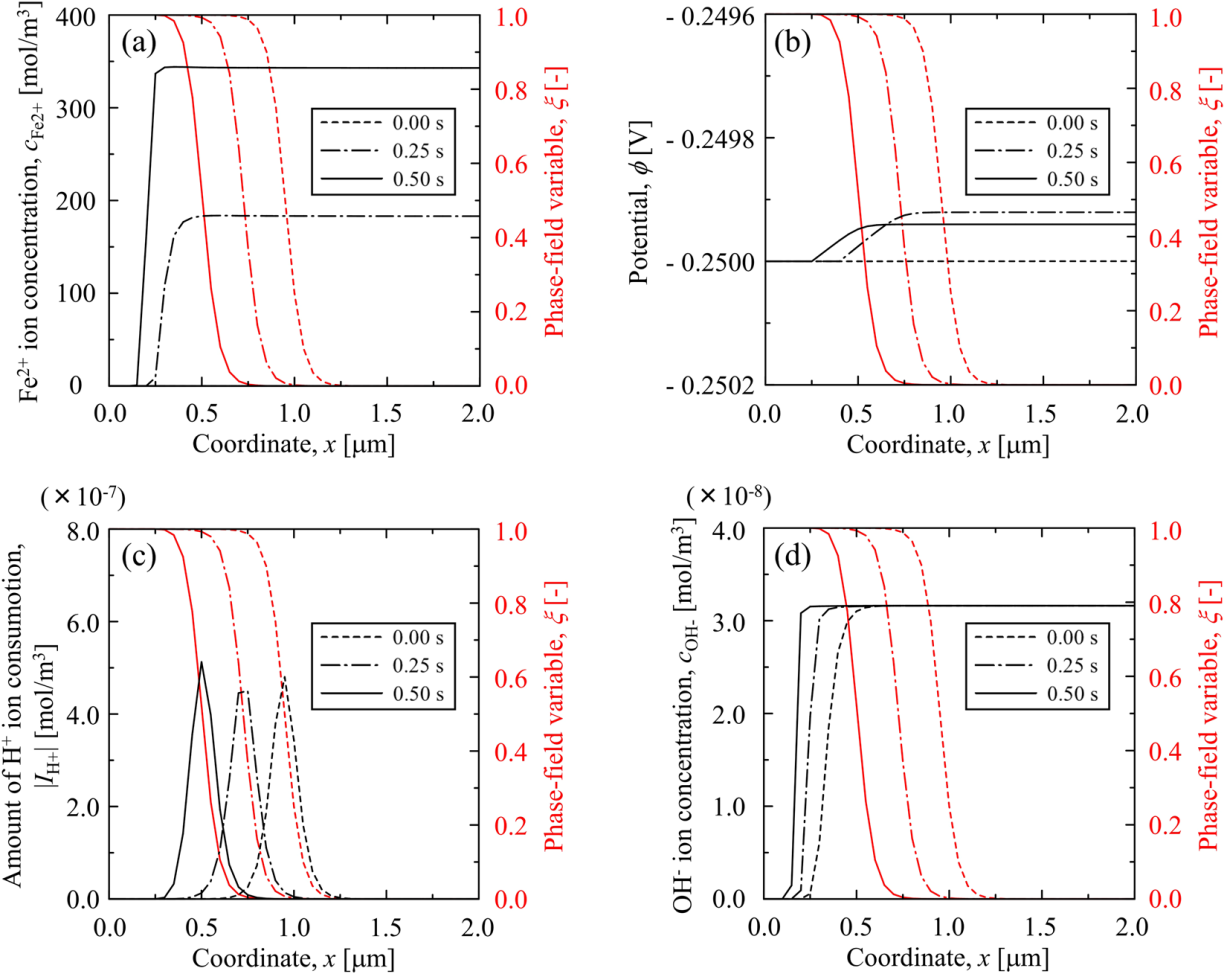
Evolutions of (**a**) Fe^2+^ ion concentration, (**b**) electrostatic potential, (**c**) consumption of H^+^ ion in the interface region, and (**d**) OH^−^ ion concentration at the pH of 2.5 and 60 °C (Case #2). In each figure, the profiles of the phase field variable are inserted to indicate the migration of iron electrode surface during the general corrosion.

In order to examine a typical result of the general corrosion simulated by the proposed PF model, the simulation result for the initial pH of 2.5 at 60 °C (Case #2) is shown in Fig. 2. As the phase-field variable, *ξ* (***r***, *t*), is defined as the local existence probability of the iron electrode: *ξ* (***r***, *t*) is 0 in the electrolyte solution and 1 in the iron electrode, the time evolution of the phase field variable shown in Fig. 2(a) describes the migration of the corroding iron surface. When the iron electrode begins to dissolve into the electrolyte solution, the Fe^2+^ ion concentration increases in the solution with *ϕ* < 1. When the dissolution of the iron electrode proceeds with time, the Fe^2+^ ion in the bulk electrolyte increases. The profile of the Fe^2+^ ion concentration in the electrolyte is almost uniform because the diffusion of the Fe^2+^ ion in the electrolyte significantly exceeds the migration of the iron electrode surface.

The variation in the electrostatic potential is shown Fig. 2(b). After the iron electrode begins to dissolve into the electrolyte solution, the potential slightly increases from the initial applied potential. Figure 2(c) describes the consumption of the H^+^ ion by the cathodic reaction in the interfacial region. It is observed that the amount of H^+^ consumption is time-independent and the variation of H^+^ ion concentration is extremely small, thereby resulting in a negligible change in the pH in the electrolyte solution. Therefore, the corrosion rate does not change during the general corrosion for 0.5 s. As shown in Fig. 2(d), the distribution of anions in the bulk electrolyte are not changed since the electrochemical reactions of Cl^−^ and OH^−^ ions are not considered in this study and the potential distribution in the electrolyte solution is uniform.

Although the corrosion rate appears as independent of the pH of the electrolyte solution, the simulation results in case #2 demonstrate that the proposed PF model captures the time-dependent migration of the iron electrode and the evolutions of the *local* ion concentrations for all species in the electrolyte solution.

**Figure 3:**
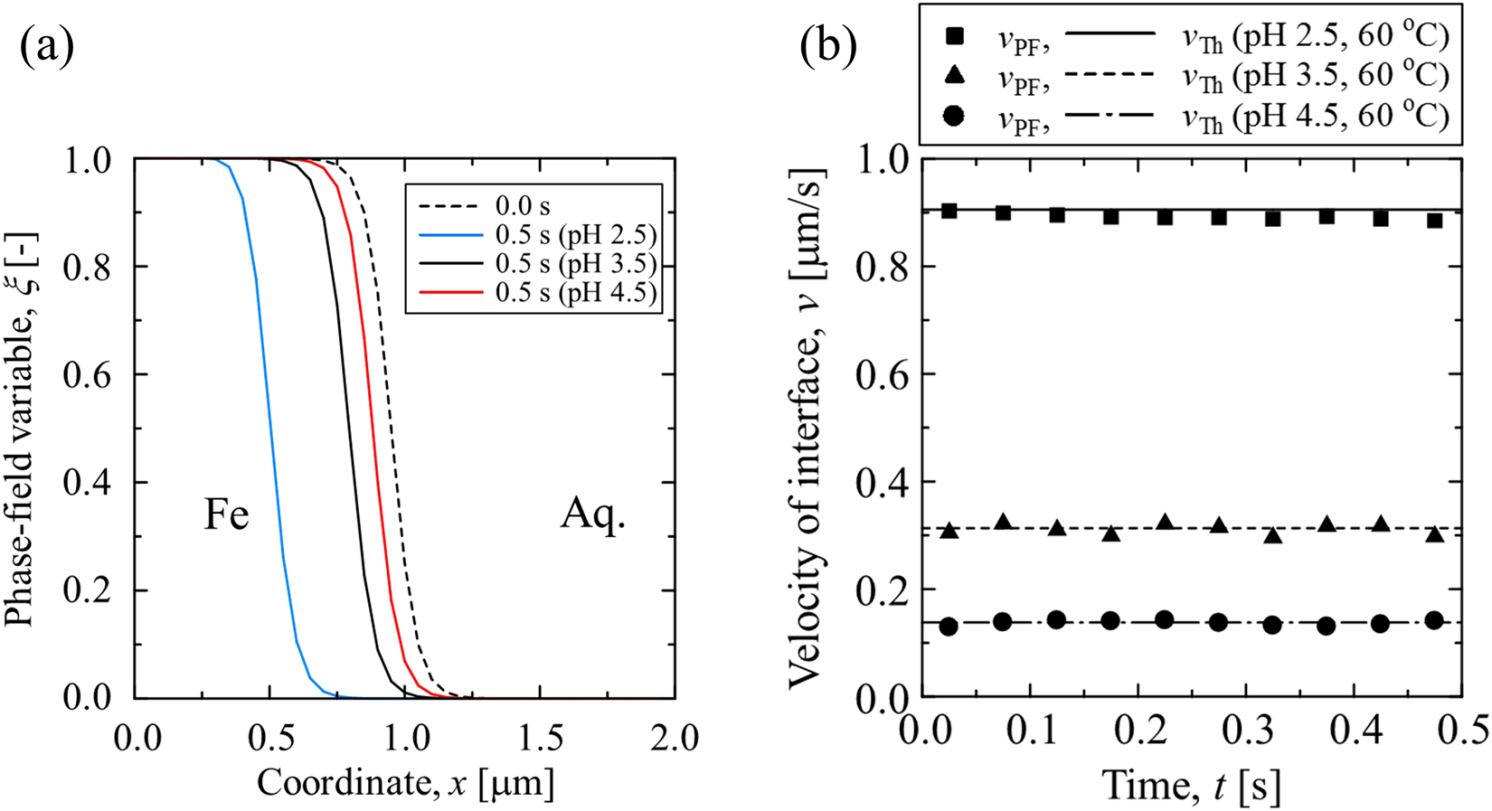
(**a**) Profiles of the phase field variable representing the position of iron electrode surface before and after the general corrosion for 0.5 s. (**b**) The interfacial migration rates calculated by the PF model, *v*_PF_, when compared with the theoretical rate, *v*_Th_ for different pH levels of the electrolyte at 60 °C (case #2, #4 and #5).

The results of the PF simulations in different cases (case #2, #4 and #5) are shown in Fig. 3 to examine the ability of the proposed PF model to capture the pH-dependent corrosion. The profiles of the phase field variable indicating the position of the iron electrode surface at 0 s and 0.5 s for different pH values are shown in Fig. 3(a). As shown in the polarization curves (Fig. 1(b–d)), the anodic current density increases with decreases in the pH of the electrolyte for the present applied potential and temperature. Therefore, as shown in Fig. 3(a), the interfacial migration rate, i.e., the corrosion rate, increases when the pH of the electrolyte decreases. The rate of the interfacial migration as calculated by the PF model, *v*_PF_, is validated by comparing it with the theoretical rate, *v*_Th_, which is calculated by Faraday’s law as follows:

20 $${v}_{{\rm{Th}}}=\frac{{i}_{a}}{2{\rho }_{{\rm{Fe}}}F}.$$

As shown in Fig. 3(b), the calculated *v*_PF_ indicates a good agreement with *v*_Th_ for all pH values.

**Figure 4:**
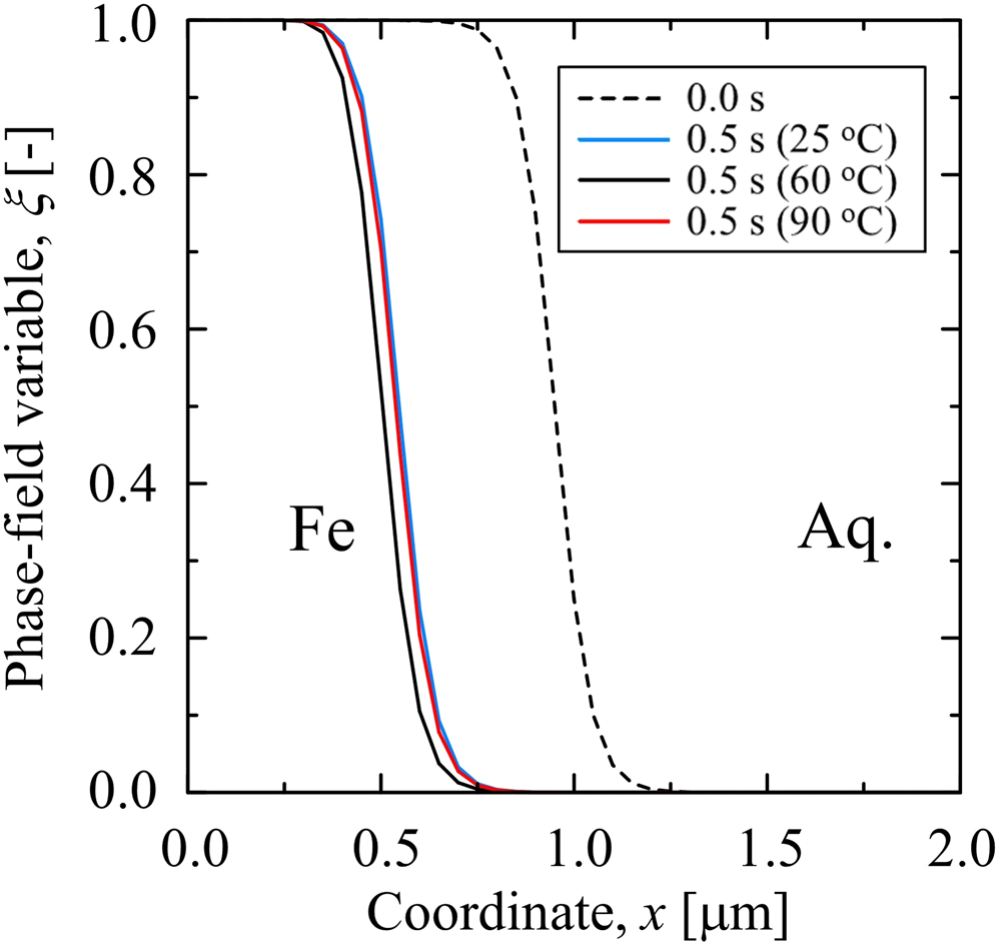
Profiles of the phase field variable indicating the position of the iron electrode surface before and after the general corrosion for 0.5 s at different temperatures and the pH of 2.5 (case #1, #2 and #3).

In order to further demonstrate that the proposed PF model can capture the effect of temperature on the corrosion rate, Fig. 4 shows the profiles of the phase field variable indicating the position of the electrode surface after 0.5 s at different temperatures (Case #1, #2 and #3) at the pH of 2.5. It is clearly observed that the corrosion rate slightly increases when the temperature increases, thereby indicating that the impact of temperature on the general corrosion rate is minor when compared with that of the pH of the electrolyte.

Based on the simulation results of the general corrosion, we demonstrate that the PF model coupled with the thermodynamic data of the electrolyte as calculated by the Corrosion Analyzer software allows us to simulate the pH- and temperature-dependent general corrosion behavior of pure iron.

### Pitting corrosion

**Figure 5:**
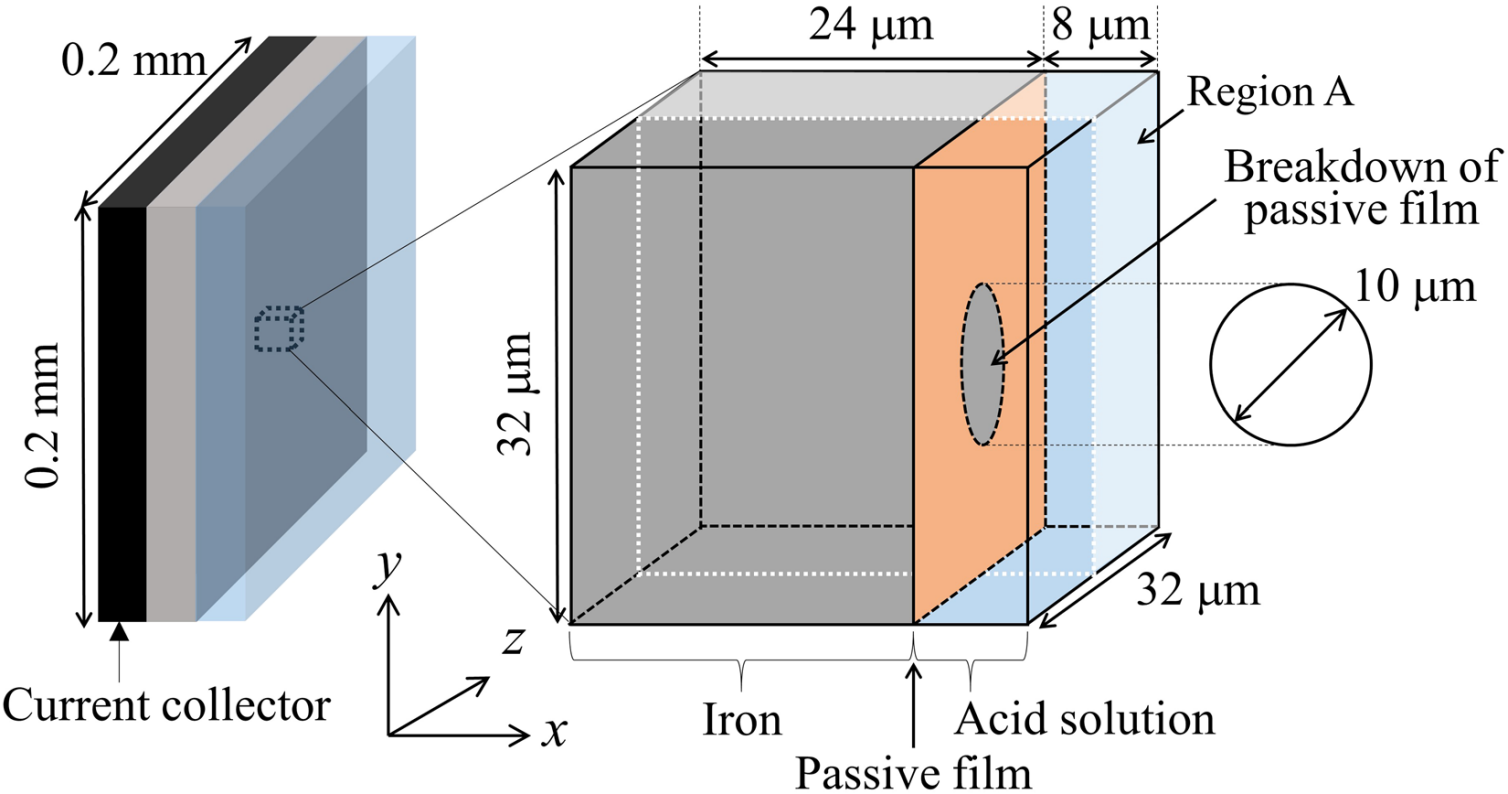
Simulation model used to simulate the pitting corrosion on a pure iron surface covered by a passive film.

The PF model used to simulate the general corrosion is subsequently applied to simulate the pitting corrosion on a pure iron sheet immersed in the acid solution. As illustrated in Fig. 5, the surface of an iron sheet with the area of 0.2 × 0.2 mm^2^ is defined as the computational domain. Although the passive cover film is not explicitly considered, we assume that the passivated film covers the top surface of the iron sheet. The propagation of a single pit commencing from a circular breakdown of the protective passive film is simulated. It is assumed that the surface of the iron sheet in the pit is always depassivated during the pitting corrosion while the film on the iron surface is fully passivated when the cathodic reaction is maintained. The initial pH of the electrolyte and the temperature are assumed as 2.5 and 60 °C, respectively. The initial concentrations of H^+^ and OH^−^ ions are calculated based on the pH and assumed as uniform in the whole electrolyte. The initial Cl^−^ ion concentration is determined such that the iron sheet is immersed into the acid solution that contains Cl^−^ ions equivalent to 1.0 M NaCl. The applied potential of the iron electrode *E* is assumed as equal to the corrosion potential and we use *E* = −420 mV irrespective of the pH of the electrolyte solution.

In the simulation of the general corrosion shown in the previous section, the mobilities, i.e., *L*_*σ*_ and *L*_*η*_, were assumed as constant, because the pH in the electrolyte did not significantly change within the simulation. In contrast, the local pH inside the pit largely varies when the pit grows. Therefore, in order to describe the variation of the pitting rate by the local pH in the electrolyte, we evaluate the mobilities as the functions of the pH. (See Methods for the details of the pH-dependent mobilities used in this study).

**Figure 6:**
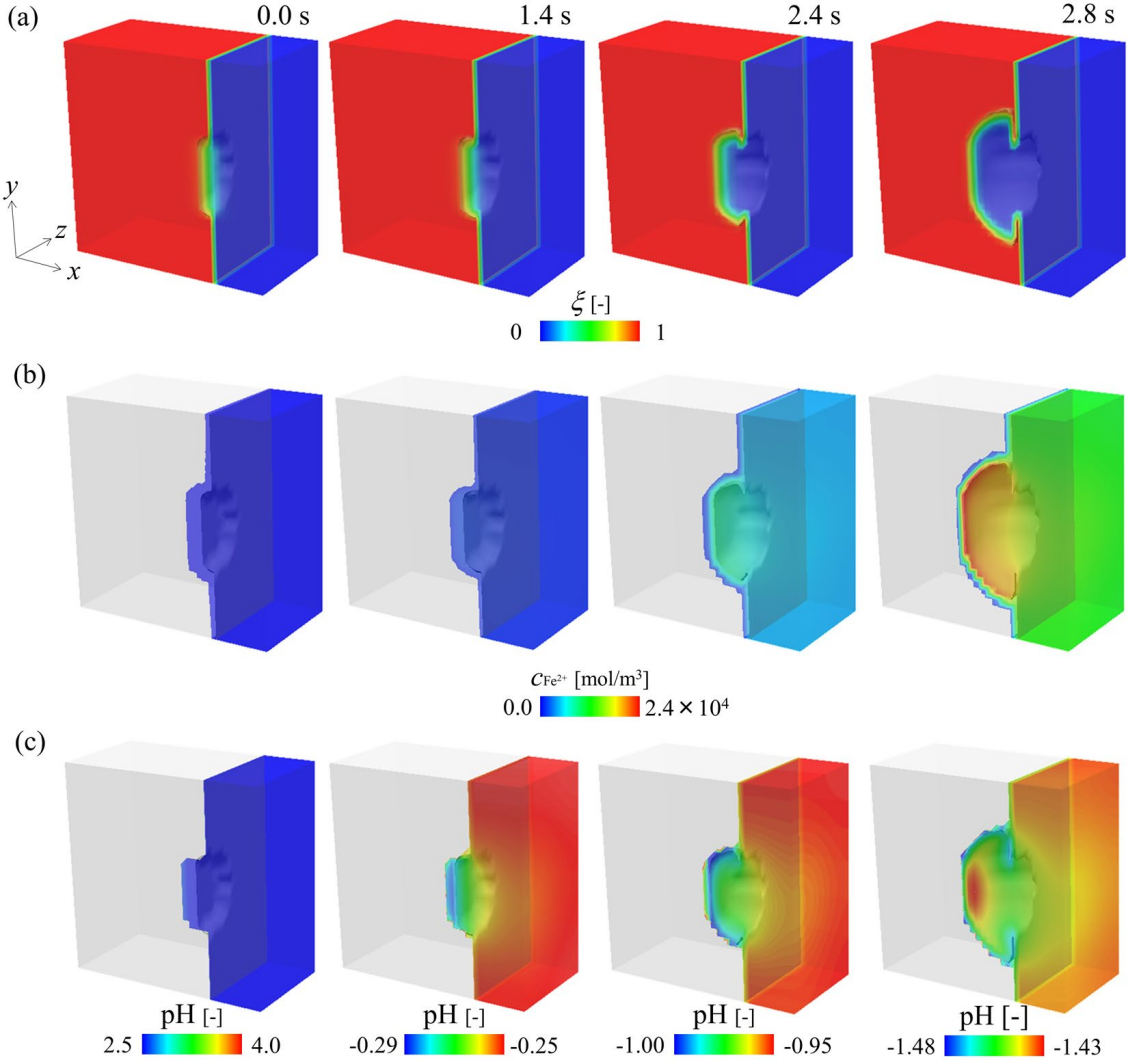
Evolutions of (**a**) phase field variable indicating the pit growth, (**b**) Fe^2+^ ion concentration, and (**c**) pH in electrolyte during the pitting corrosion for 2.4 s.

**Figure 7:**
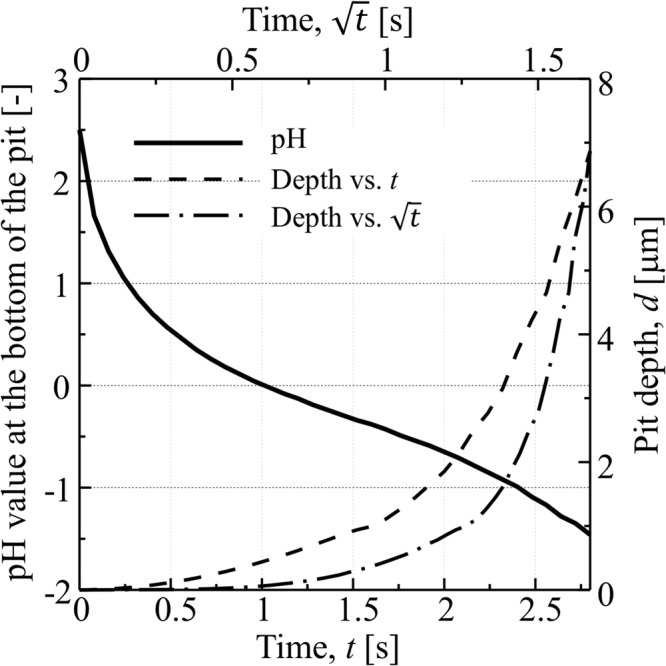
Variations in the pit depth and the local pH at the bottom of the pit during the pitting corrosion.

**Figure 8:**
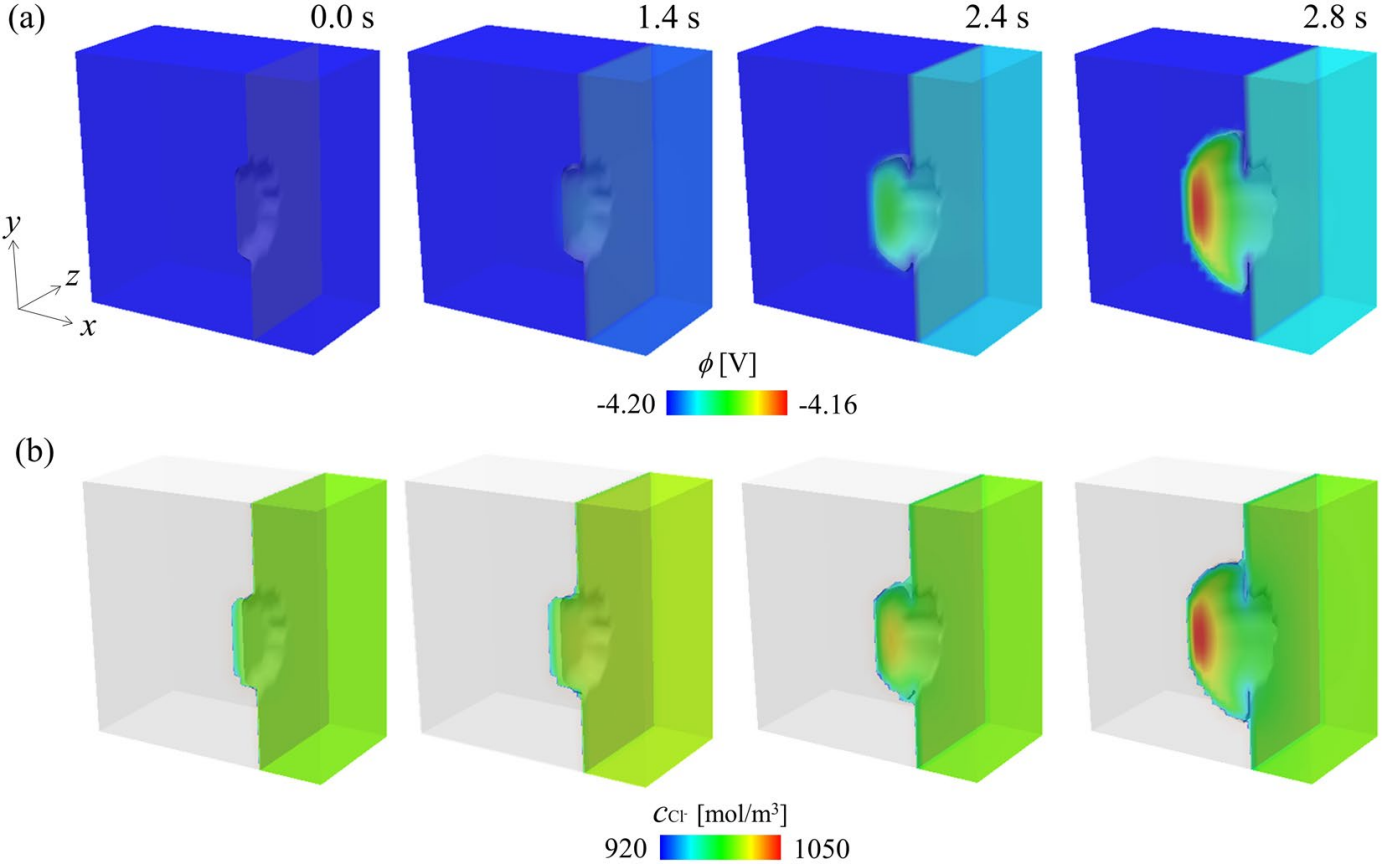
Evolutions of (**a**) electrostatic potential and (**b**) Cl^−^ ion concentration during the pitting corrosion for 2.4 s.

Figure 6(a) shows the evolution of the phase field variables on a cross section passing through a pit (Region A indicated in Fig. 5), indicating the morphological change of the pit. The pit begins to grow into the iron electrode from the circular breakdown of the passive film. In the early stage (approximately up to 1.4 s), the pit slowly migrates to the inside of the iron electrode. Subsequently, the pit undercuts the iron electrode beneath the surface protected by the passive film and results in the anisotropic growth of the pit. When the pit grows, the Fe^2+^ ion concentration increases in the solution within the pit due to the dissolution of the iron electrode. In the later stage, as shown in Fig. 6(b), the Fe^2+^ ion flows from the pit mouth to the bulk electrolyte. In contrast with the dissolution of iron, the reduction of H^+^ ion occurs inside the pit. Therefore, as shown in Fig. 6(c), the local pH in the electrolyte exhibits a significant decrease from the initial value of 2.5 to the negative value for 1.4 s. Figure 7 shows the variations in the pit depth and the local pH at the bottom of the pit. The local pH significantly decreases in the early stage (approximately 0.35 s) although the growth of the pit is extremely slow. As shown in Methods, when the pH decreases as low as zero or a negative value at 1.0 s, the anodic current density increases such that it is approximately 300 times higher than the initial value at the pH of 2.5. Therefore, as shown in Fig. 6(c), the pit rapidly propagates after 1.4 s at which the pH in the pit reaches a negative value. During the pit growth, the distribution of the pH inside the pit is not uniform. As shown in Fig. 6(c), the pH exhibits a maximum at the bottom of the pit and a minimum near the pit mouth because the H^+^ ion migrates from the pit front to the mouth due to the electrostatic potential gradient shown in Fig. 8(a). In the previous study on the finite element analysis of the localized corrosion of aluminum alloy^32^ reported that the pH decreases in the direction of the pit depth. However, the pH distribution in a pit strongly depends on the existence of passive film which covers the pit. In the case of the pit covered by the passive film, the transportation of hydrogen ion from the inside to the outside of the pit is prevented by the passive film, resulting that the pH increases in the depth direction. The phase-field model proposed in this paper also reveals that the pH in the pit decreases in the depth direction if no passive film covers the pit (see Section 2 of Supplemental information). The inhomogeneous pH distribution in the pit leads to anisotropic pit growth.

Furthermore, the evolut1ion of Cl^−^ ion concentration during the pit growth is shown in Fig. 8(b). Although both production and consumption of Cl^−^ ion are not considered in the study, the simulations reveal that the Cl^−^ ion migrates to the bottom of the pit due to the gradient of the electrostatic potential and results in the aggregation of Cl^−^ ions at the growing pit front. Based on the above-mentioned results, we demonstrate that the proposed PF model captures the key features of the pitting corrosion including the morphological change in the propagating pit, variations in the local pH inside the pit that strongly affects the anisotropic pit propagation, and ionic concentrations (especially the aggregation of Cl^−^ ion).

According to the previous experimental observations of pit growth in stainless steels^33,34^, the pit depth increases linearly with the square root of time ($\sqrt{t}$) under diffusion controlled pitting corrosion. However, as shown in Fig. 7, the variation of pit depth calculated by the present phase-field model is non-linear. Although it is not easy to compare the pit growth rate calculated in this study with that experimentally measured in the exactly same condition, the calculated rate of the pit growth after 2.5 s where the growth is almost linear to $\sqrt{t}$ is much higher than those reported in the previous experimental studies^33,34^. In order to quantitatively compare the calculated pit growth rate with experimentally measured one, we need to consider the formation of passive film which covers the pit and the repassivation within the growing pit.

Before closing this section, we further note the limitation of the phase-field model proposed in this paper. In this study, we consider the pH-dependent mobility (Eqs (21) and (22)) and the anodic current density (Eq. (27)) obtained by the Corrosion Analyzer, but the other parameters are also influenced by pH values of the electrolyte solution. For instance, the pH-dependent hydrogen ion (H^+^) activity coefficient is an important one to obtain the corrected pH values especially at high H^+^ concentrations (see Fig. 3 in Supplemental information), for simulating the corrosion behavior of iron more quantitatively. Although not considered in this work, the pH-dependent parameters, such as the H^+^ activity, the electric conductivity and the ion diffusion coefficients, should be implemented into the phase-field model in our future work.

## Conclusions

The new PF model was proposed to simulate the corrosion behavior of ferrous metals that strongly depends on the pH of the electrolyte solution. The present PF model was formulated based on the Bockris’s mechanism to implement the ability to describe the pH-dependence of the corrosion process. We also proposed a simulation methodology to incorporate the thermodynamic data of the electrolyte solution obtained by using the Corrosion Analyzer software with respect to the PF model. The proposed PF model was applied to the simulations of two corrosion problems, namely general corrosion and pitting corrosion in a pure iron immersed in the acid solution.

The simulation results of the general corrosion demonstrated that the PF model successfully captured the migration of the corroding iron surface, local changes in ionic concentrations, and electrostatic potential in the electrolyte during the general corrosion. We investigated the effects of the pH of the bulk electrolyte and temperature on the corrosion rate. The results demonstrated that the PF model captured the increase in the corrosion rate with decreases in the pH value and increases in the temperature. Furthermore, the PF simulation of the pitting corrosion revealed that the proposed PF model successfully simulated the key features of the pitting corrosion including the morphological change in the growing pit that depends on the local pH in the pit, evolution of the electrostatic potential, and concentration of the Cl^−^ ion at the growing pit front. In future work, in order to simulate the corrosion behavior in iron more quantitatively, we need to consider not only the pH-dependent anodic current density, but also the pH-dependent other parameters: H^+^ activity coefficients, electric conductivity, and ion diffusion coefficients.

## Methods

### Simulation condition for general corrosion

The length of the computational domain is 2.0 μm. The domain is divided by 40 uniform finite difference grids. The spacing between the grids is *Δx* = 0.05 μm. The thickness of the diffuse interface is set as *δ* = 4*Δx* = 0.2 μm. The time increment is *Δt* = 2 × 10^−7^ s. The iron electrode with a thickness of 1.0 μm is placed at the left end of the domain, and other part is set as the aqueous solution. The initial concentrations of H^+^ and OH^−^ ions in the electrolyte solution are set as constant at the values calculated from the pH. The initial concentration of Cl^−^ ion is set based on the charge neutrality in the solution. The initial distributions of the ions are calculated by solving the Nernst–Planck equation with the fixed interface. The zero flux boundary condition is applied for the phase-field variable and the ion concentrations at both ends of the computational domain.

In the simulation of the general corrosion, *I*_Fe2+_ and *I*_H+_ are calculated based on Eqs (1) and (2) as follows:

21 $${I}_{{{\rm{Fe}}}^{2+}}={\rho }_{{\rm{Fe}}}|{\rm{\Delta }}\xi |$$

22 $${I}_{{{\rm{H}}}^{+}}=-\,2{\rho }_{{\rm{Fe}}}\frac{{i}_{c}}{{i}_{a}}|{\rm{\Delta }}\xi |$$

where *i*_a_ and *i*_c_ denote the anodic and cathodic current densities, respectively, as calculated by Corrosion Analyzer software and listed in Table 2. The mobilities of the interface, i.e., *L*_*σ*_ and *L*_*η*_, are assumed as constant in the simulations of the general corrosion. We determine the mobilities such that the dissolution rate of the iron electrode in the initial stage of the general corrosion is consistent with the anodic current density, *i*_a_. The values of the mobilities used in the simulation of the general corrosion are listed in Table 3.

**Table 2:** Combinations of the pH of the electrolyte solution and temperature examined in the PF simulations of the general corrosion.

Case #	pH	Temperature, *T* [°C]	Anodic current density, *i*_a_ [A/m^2^]	Cathodic current density, *i*_c_ [A/m^2^]
1	2.5	25	220.0	0.1247
2	2.5	60	244.6	0.4223
3	2.5	90	227.8	0.8710
4	3.5	60	84.63	0.04632
5	4.5	60	37.23	0.004797

Anodic and cathodic current densities at the applied potential of *E* = −250 mV vs. SHE in each case are calculated by using Corrosion Analyzer software.

**Table 3:** Phase-field mobilities for the interfacial part and reaction part in each case.

Case #	pH	Temperature, *T* [°C]	Phase-field mobility for the interfacial part, *L*_*σ*_ [m^3^/(J·s)]	Phase-field mobility for the reaction part, *L*_*η*_ [/s]
1	2.5	25	5.86 × 10^-15^	1.17 × 10^6^
2	2.5	60	2.13 × 10^-13^	4.26 × 10^6^
3	2.5	90	4.58 × 10^-14^	9.15 × 10^6^
4	3.5	60	7.38 × 10^-15^	1.48 × 10^5^
5	4.5	60	3.25 × 10^-15^	6.49 × 10^3^

In a numerical simulation, we calculate Eqs (15) and (17) by a second-order finite difference scheme for space and a first-order forward Euler-type finite difference method for time on the regular computational grid. Equation (19) is calculated by the Successive Over-Relaxation (SOR) method. Note that the same discretization methods are used in the simulation of the pitting corrosion, and no fatal error does not occur during the numerical simulations using the finite difference schemes. The simulation flow chart which explains the coupling between the calculations of the phase-field variable, the ion concentration and the electrostatic potential is shown in Section 3 of Supplemental information.

### Acceleration of general corrosion rate

The rate of the general corrosion is generally the order of a few mm/year. Thus, we artificially accelerate the rate of iron dissolution by decreasing the molar density of iron to check whether the proposed PF model captures the migration of the iron electrode surface and the evolution of the ionic concentrations in the solution. The acceleration corresponds to increases in the current density without using any unrealistic parameters as follows: Based on Faraday’s second law, the velocity of the migrating iron electrode surface is given as follows:

23 $${v}_{d}=\frac{{i}_{a}}{2{\rho }_{{\rm{Fe}}}F}$$

The corrosion rate increases with increases in the anodic current density, *i*_a_,. However, *i*_a_ also governs the production and the consumption of the ions at the electrode surface. Thus, instead of increasing *i*_a_, we accelerate the rate of iron dissolution by decreasing the molar density of iron, *ρ*_Fe_,. For example, if *ρ*_Fe_ is decreased to *ρ*_Fe_^*^ = *ρ*_Fe_/*k*, where *k* > 1, then the accelerated corrosion rate *v*_d_^*^ is expressed as follows:

24 $${v}_{d}^{\ast }=\frac{{i}_{a}}{2{\rho }_{{\rm{Fe}}}^{\ast }F}=\frac{k{i}_{a}}{2{\rho }_{{\rm{Fe}}}F}$$

In this study, we use *k* = 100.

### Simulation condition for pitting corrosion

The size of the computational domain is set as 32 × 32 × 32 μm^3^. The domain is divided by using 32 × 32 × 32 regular finite difference grids. We aim to simulate the growth of a pit whose size is a few micrometers. Therefore, the grid spacing is 1 μm. The thickness of the interface is set as *δ* = 3Δx = 3 μm. The thickness of the passive film is assumed as equal to a single finite difference grid. The zero Neumann boundary conditions at *x* = 32 μm are applied for Fe^2+^ and H^+^ ion concentration fields. With respect to other ion concentrations and the phase field variable, we applied the zero Neumann condition at *x* = 0 μm and the periodic boundary condition along the *x* and *z* directions. It should be noted that the acceleration of the corrosion rate used in the general corrosion simulation is not used in the simulation of the pitting corrosion.

In the simulation of the pitting corrosion, we evaluate the pH-dependent mobilities by using the following equations:

25 $${L}_{\sigma }({\rm{pH}})={L}_{\sigma 0}\frac{{i}_{a}({\rm{pH}})}{{i}_{a0}}$$

26 $${L}_{\eta }({\rm{pH}})={L}_{\eta 0}\frac{{i}_{a}({\rm{pH}})}{{i}_{a0}}$$

where *i*_a0_, *L*_*σ*0_, and *L*_*η*0_ denote the anodic current density and the mobilities at the initial pH of 2.5. Additionally, *i*_a_(pH) denotes the anodic current density at the corrosion potential and is assumed as dependent of the pH of the electrolyte solution. Furthermore, *i*_a_(pH) is calculated by Corrosion Analyzer software and approximated by the following polynomial functions:

27 $${i}_{a}({\rm{pH}})=\{\begin{array}{c}-60792{{\rm{pH}}}^{4}-238835{{\rm{pH}}}^{3}-227116{{\rm{pH}}}^{2}-92012{\rm{pH}}-4712.2\,(\,-\,2.4\le {\rm{pH}}\le -{\rm{1.0}})\\ \begin{array}{c}-34543{{\rm{pH}}}^{3}-6705.3{{\rm{pH}}}^{2}-7036.4{\rm{pH}}+3949.4\,(\,-\,1.0\le {\rm{pH}}\le {\rm{0.0}})\\ \begin{array}{c}1281.9{{\rm{pH}}}^{2}-3982.9{\rm{pH}}+3974.9\,(0.0\le {\rm{pH}}\le {\rm{1.0}})\\ 471.25{{\rm{pH}}}^{2}-2384.3{\rm{pH}}+3145.1\,(1.0\le {\rm{pH}}\le {\rm{2.5}})\end{array}\end{array}\end{array}$$

**Figure 9:**
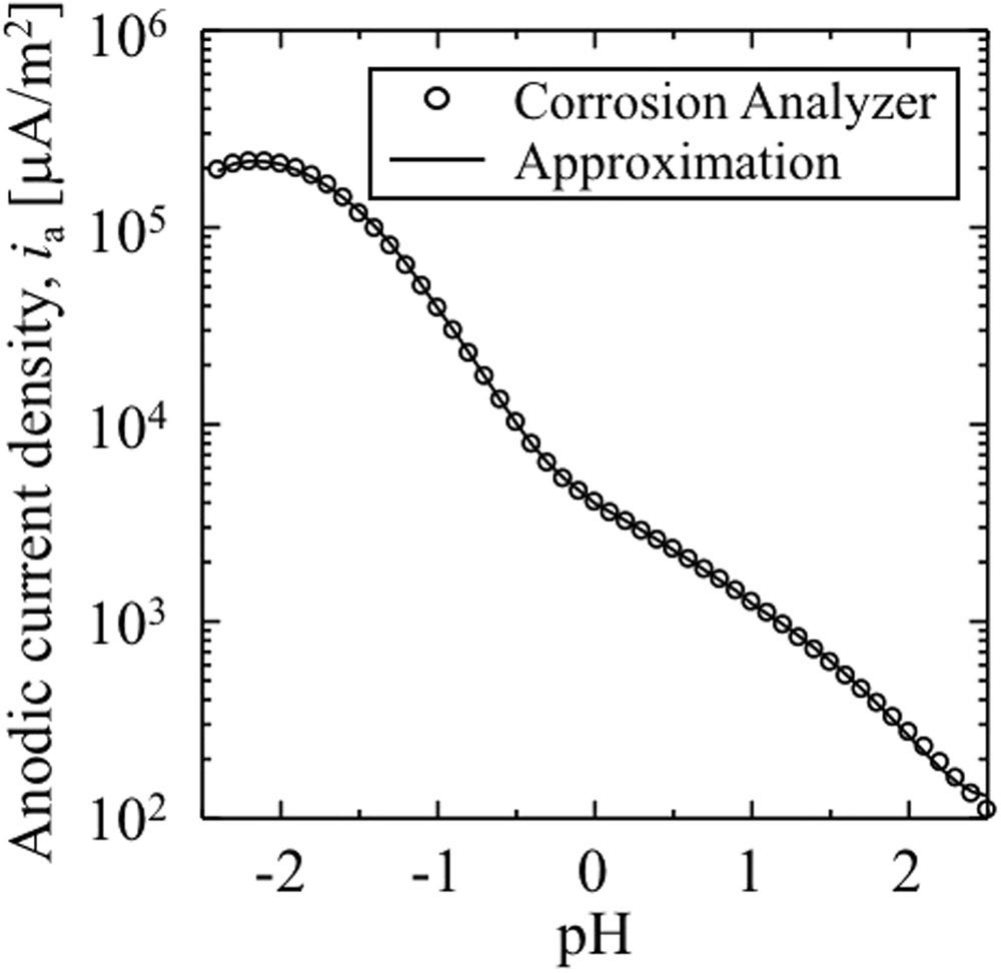
Anodic current density evaluated as a function of the pH of the acid solution at 60 °C. White circles and black line denote the anodic current density at the corrosion potential calculated by Corrosion Analyzer and the approximated curve used in the simulation of the pitting corrosion, respectively.

Figure 9 shows the data of *i*_a_(pH) as obtained by the Corrosion Analyzer software and the approximated curve used in the pitting corrosion simulation.

In the pitting corrosion simulation, *I*_Fe2+_ and *I*_H+_, used for Eq. (17) are calculated based on Eqs (1) and (6) as follows:

28 $${I}_{{{\rm{Fe}}}^{2+}}={\rho }_{{\rm{Fe}}}|\Delta \xi |$$

29 $${I}_{{H}^{+}}=2{\rho }_{{\rm{Fe}}}|\Delta \xi |$$

It should be noted that the consumptions of Fe^2+^ and Cl^−^ ions due to the hydrolysis of Fe^2+^ ion and FeCl_2_ in the pit are not considered because we assumed here that the impact of the generation of Fe^2+^ ion by the iron dissolution on the corrosion rate is much larger than that of the consumption of Fe^2+^ ion due to the hydrolysis of Fe^2+^ ion. In the simulation of the pitting corrosion, the equilibrium constants for the Bockris mechanism, *K*_1_ and *K*_3_, are set as 1.0 × 10^6^. Since the conductivity of the electrolyte solution, *σ*^s^, depends on the ion concentrations and the pH of the solution, we assumed it as *σ*^s^ = 100 [S/m] according to the calculation results obtained by the Corrosion Analyzer.

## Electronic supplementary material

Supplemental information
