# Supplementary material for: Phase-field modeling for pH-dependent general and pitting corrosion of iron
Source: Sci Rep. 2018 Aug 24;8:12777. doi: 10.1038/s41598-018-31145-7 (PMC6109116; doi:10.1038/s41598-018-31145-7)
Supplement: Supplementary file 1 — Supplemental information [file 41598_2018_31145_MOESM1_ESM.docx]

**Supplementary information for**

**Phase-field modeling for pH-dependent general and pitting corrosion of iron**

Chisa Tsuyuki^1^, Akinori Yamanaka^2*^, & Yasushi Ogimoto^3^

^1^ Department of Mechanical Systems Engineering, Graduate School of Engineering, Tokyo University of Agriculture and Technology, 2-24-16, Naka-cho, Koganei-shi, Tokyo 184-8588, Japan

^2^ Division of Advanced Mechanical Systems Engineering, Institute of Engineering Tokyo University of Agriculture and Technology, 2-24-16, Naka-cho, Koganei-shi, Tokyo 184-8588, Japan

^3^ Advanced Technology Laboratory, Fuji Electric Co., Ltd., 1, Fuji-machi, Hino-city, Tokyo 191-8502, Japan

^*^Correspondence and requests for materials should be addressed to A. Y. (email: a-yamana@cc.tuat.ac.jp)

**1. Formulation of phase-field model**

The PF model proposed in this study is inspired by the nonlinear PF model originally proposed by Chen et al.^1^. We derive the time evolution equation of the phase field variable, and this describes the migration of iron electrode surface by the corrosion based on Eq. (4) that corresponds to the rate determining process of the Bockris mechanism. The formulation of the PF model proposed in this study is shown below.

The electrochemical potential of the ionic species i is given as follows:

|  | (S1) |
| --- | --- |

where *R* denotes the gas constant, *T* is temperature, and *φ* denotes the electrostatic potential. $\mu_{i}^{\Theta}$, *a_i_* and *z*_i_ denote the reference chemical potential, activity, and valence of species i, respectively. By using Eq. (S1), the electrochemical potential for different species related to the rate determining process (Eq. (4)) of the Bockris mechanism are expressed as follows:

|  | (S2) |
| --- | --- |
|  | (S3) |
|  | (S4) |

where *φ*_s_ and *φ*_e_ denote the electrostatic potential in the electrode and the solution, respectively.

At the equilibrium state of the chemical reaction given by Eq. (3), is satisfied. Therefore, the equilibrium potential of the electrode, *E*_eq_, is expressed as follows:

|  | (S5) |
| --- | --- |

where *E*^^ denotes the standard potential of the electrode and given as follows:

|  | (S6) |
| --- | --- |

With respect to the out of equilibrium state, the total overpotential, *η*, is defined as follows:

|  | (S7) |
| --- | --- |

where ** denotes the overpotential difference for changing Fe to Fe^2+^ by dissolving the iron electrode. *G* denotes the total free energy of the system.

Conversely, the total overpotential is also given by the Nernst equation as follows:

|  | (S8) |
| --- | --- |

where *E* denotes the potential of an electrode versus a reference, and *η*_a_ and *η*_c_ denote the activation and concentration overpotentials, respectively. Given that *η* = *η*_a_ + *η*_c_ and Eq. (S8), the overpotentials are expressed as follows:

|  | (S9) |
| --- | --- |
|  | (S10) |

It is assumed that *K*_1_ and *K*_3_ denote the equilibrium constants of the chemical reaction given by Eq. (3) and (5), respectively, and the activities of FeOH^+^ and (FeOH)_ads_, i.e., *a*_FeOH+_ and *a*_(FeOH)ads_, are given as follows:

|  | (S11) |
| --- | --- |
|  | (S12) |

By using Eqs. (S11) and (S12), Eq. (S10) is reduced as follows:

|  | (S13) |
| --- | --- |

By using Eqs. (S7), (S8), (S9), and (S13), the total reaction rate, *R*_e_, is given by the Butler–Volmer kinetics as follows:

|  | (S14) |
| --- | --- |

where *α* denotes the transfer coefficient. *R*_0_ denotes the exchange reaction rate at the equilibrium state that is given as follows:

|  | (S15) |
| --- | --- |

where *k*_0_ denotes the reaction constant, and *γ *_t_ denotes the activity coefficient at the transition state.

We follow a method similar to Chen et al.^1^ and consider the total reaction rate that denotes the rate of the interfacial migration. This is determined by the interfacial free energy, and the electrochemical reaction, *R*_e_, is defined as the sum of these two contributions as follows:

|  | (S16) |
| --- | --- |

with

|  | (S17) |
| --- | --- |
|  | (S18) |

We assume that that *R_ _* significantly exceeds *R_σ_* at the non-equilibrium state, *x* >> *y*, and thus we expand Eq. (S16) by the Taylor expansion as follows:

|  | (S19) |
| --- | --- |

with

|  | (S20) |
| --- | --- |

and

|  | (S21) |
| --- | --- |

If the interfacial mobility is defined as follows:

|  | (S22) |
| --- | --- |

Then, Eq. (S20) is expressed as follows:

|  | (S23) |
| --- | --- |

Given the definition of activity, Eq. (S23) is reduced as follows^1^:

|  | (S24) |
| --- | --- |

where *g’*(**) denotes the double well potential function. Additionally, we substitute Eqs. (S15) and (S17) into Eq. (S21) and obtain the electrochemical reaction part of the reaction rate given as follows:

|  | (S26) |
| --- | --- |

where *L_η_* is also the interfacial mobility and is given as follows:

|  | (S27) |
| --- | --- |

Finally, the migration rate of the interface is equivalent to the total reaction rate given by Eq. (S16), and thus the time evolution equation of the phase field variable is expressed as follows:

|  | (S28) |
| --- | --- |

where *L*(**) is an interpolating function that indicates that the electrochemical reaction occurs only at the surface of the electrode and is given as follows:

|  | (S29) |
| --- | --- |

It should be noted that although *η* = *η*_a_ + *η*_c_ is considered, we assume that the contribution of *η*_a_ on the total reaction rate significantly exceeds that of *η*_c_ in the study. Therefore, the total overpotential *η* is approximated as follows:

|  | (S30) |
| --- | --- |

**2. Effect of passive film on pH distribution**

In order to investigate the effect of passive film which covers the pit mouth on the pH distribution inside the pit, the pitting corrosion is simulated with and without the passive film. **Figure 1** compares the distributions of pH inside the pit for the case with and without the passive film. It is clearly seen in **Fig. 1**(b) that the pH in the pit decreases along the depth direction. This pH distribution shows a qualitative agreement with that reported by the previous study by Xiao et al.^32^.


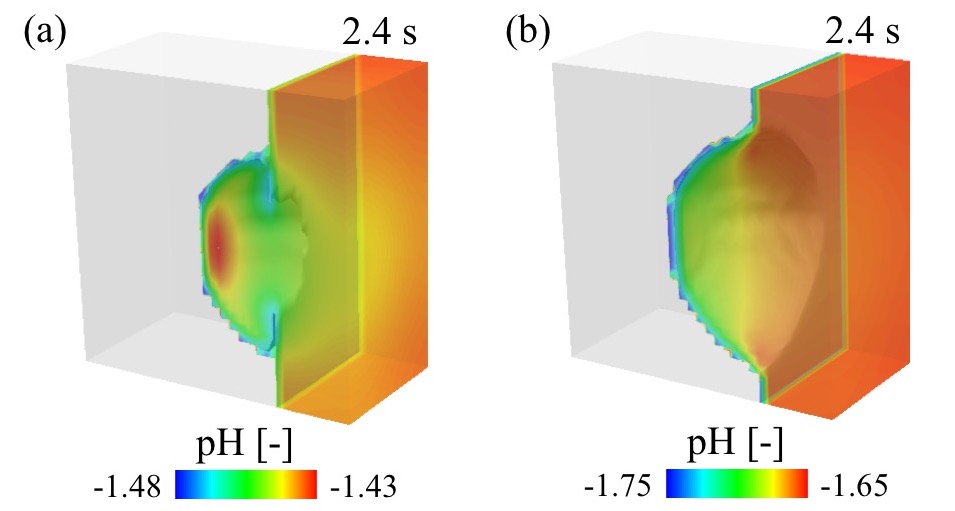


**Fig. 1** Distribution of pH in the pit (a) covered by the passive film which is same as Fig. 6(c) in the paper and (b) without the passive film after the pitting corrosion for 2.4 s.

**3. Simulation flow chart**


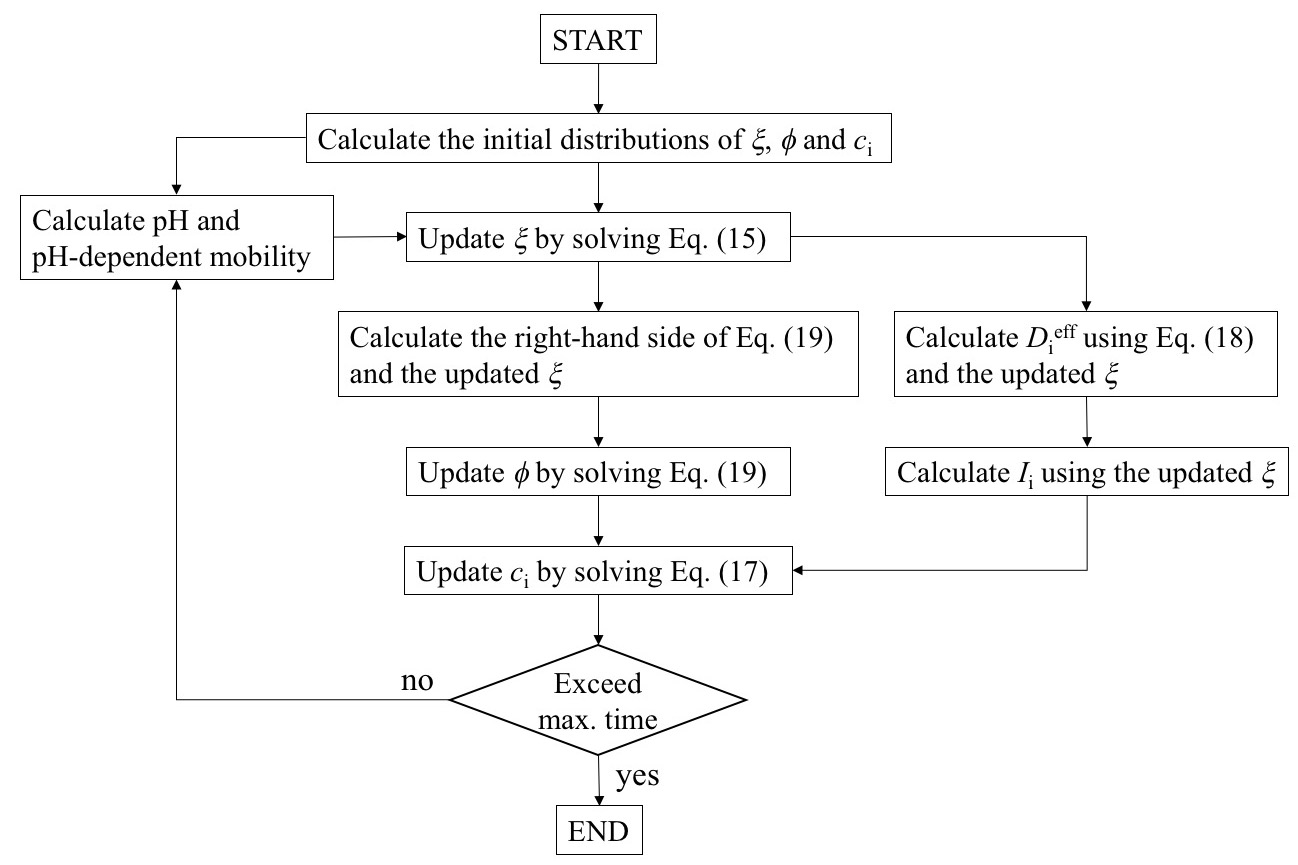


**Fig. 2** Flow chart showing the sequence of a corrosion simulation using the phase-field model proposed in this paper.

**4. Influence of hydrogen activity coefficient on pH**

| 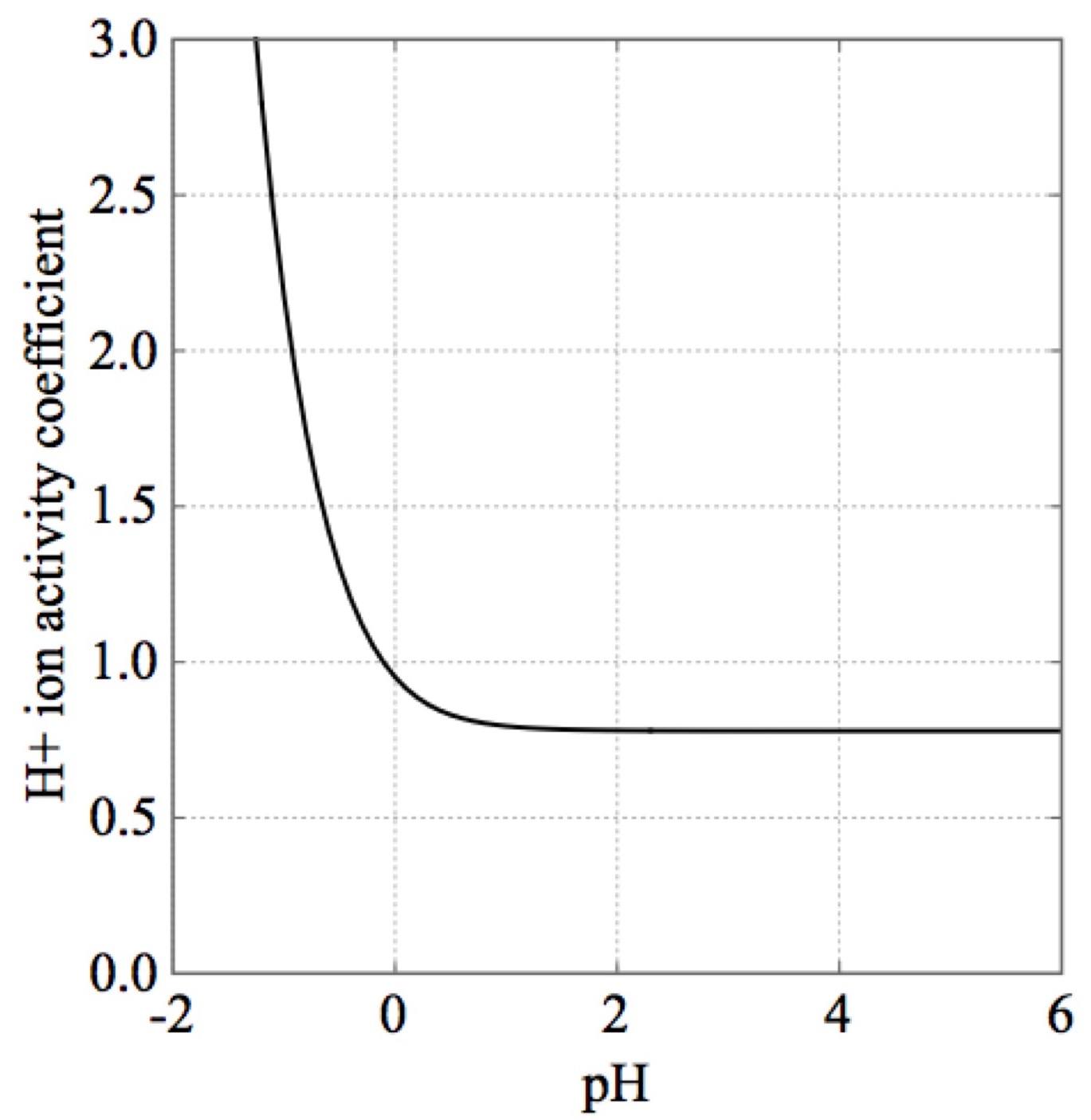 | 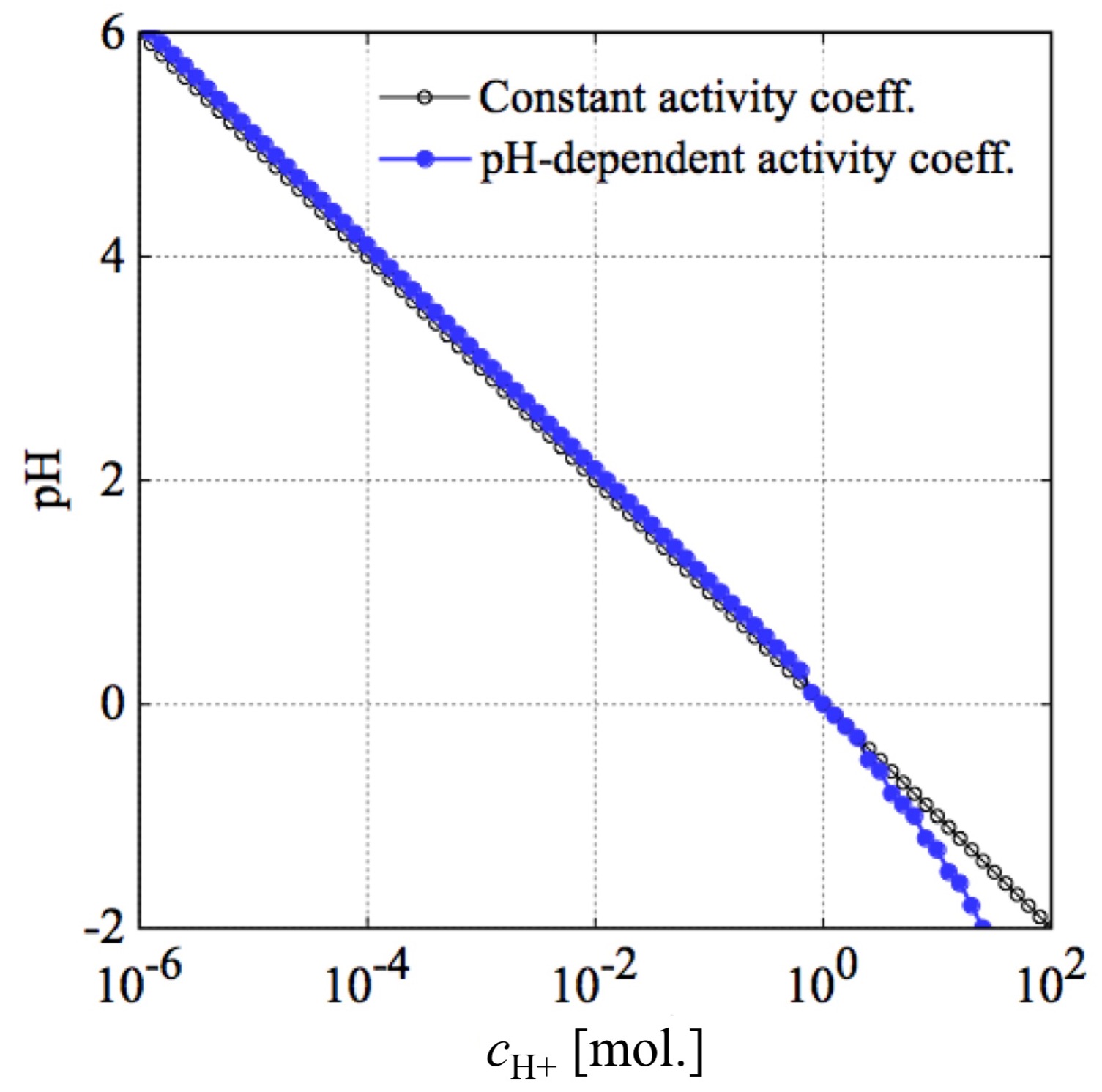 |
| --- | --- |
| (a) | (b) |

**Fig. 3** Variations of (a) the hydrogen ion activity coefficient as a function of pH calculated by Corrosion Analyzer (*T*=60 ^o^C, 1M NaCl) and (b) pH as a function of hydrogen ion concentration, *c*_H+_, using the constant hydrogen ion activity coefficient (*f*_H+_ = 1) and the pH-dependent one shown in (a).

**References**

1. Chen, L., Zhang, H. W., Liang, L. Y., Liu, Z., Qi, Y., Lu, P., Chen, J., & Chen, L.-Q. Modulation of dendritic patterns during electrodeposition: A nonlinear phase-field model. *Journal of Power Sources* **300** 376–385 (2015).
